# Common genetic variants associated with Parkinson’s disease display widespread signature of epigenetic plasticity

**DOI:** 10.1038/s41598-019-54865-w

**Published:** 2019-12-05

**Authors:** Amit Sharma, Naoki Osato, Hongde Liu, Shailendra Asthana, Tikam Chand Dakal, Giovanna Ambrosini, Philipp Bucher, Ina Schmitt, Ullrich Wüllner

**Affiliations:** 1 Department of Neurology, University Clinic Bonn, Bonn, Germany; 2 Department of Ophthalmology, University Clinic Bonn, Bonn, Germany; 3 Department of Bioinformatics Engineering, Osaka University, Osaka, Japan; 4 State Key Laboratory of Bioelectronics, Southeast University, Nanjing, China; 5 Drug Discovery Research Centre (DDRC), Translational Health Science and Technology Institute (THSTI), Haryana, 121001 India; 6 Genome & Computational Biology Lab, Department of Biotechnology, Mohanlal Sukhadia University, Udaipur, 313001 Rajasthan India; 7 EPFL and Swiss Institute of Bioinformatics, Lausanne, Switzerland; 8 German Center for Neurodegenerative Diseases (DZNE), Bonn, Germany

**Keywords:** Neuroscience, Epigenetics and plasticity

## Abstract

Parkinson disease (PD) is characterized by a pivotal progressive loss of substantia nigra dopaminergic neurons and aggregation of *α*-synuclein protein encoded by the *SNCA* gene. Genome-wide association studies identified almost 100 sequence variants linked to PD in *SNCA*. However, the consequences of this genetic variability are rather unclear. Herein, our analysis on selective single nucleotide polymorphisms (SNPs) which are highly associated with the PD susceptibility revealed that several SNP sites attribute to the nucleosomes and overlay with bivalent regions poised to adopt either active or repressed chromatin states. We also identified large number of transcription factor (TF) binding sites associated with these variants. In addition, we located two docking sites in the intron-1 methylation prone region of *SNCA* which are required for the putative interactions with DNMT1. Taken together, our analysis reflects an additional layer of epigenomic contribution for the regulation of the *SNCA* gene in PD.

## Introduction

Parkinson’s disease (PD) is a heterogeneous age-associated incurable neurodegenerative syndrome, occurring in both sporadic and familial forms. Although phenotypically diverse, the defining pathological hallmark of PD is the loss of midbrain dopaminergic neurons in the substantia nigra pars compacta. Surviving dopaminergic neurons display characteristic extensive aggregate confirmations (Lewy bodies, LB), which among other proteins contain alpha-synuclein (α-SYN). Although it is unclear why exactly neurons degenerate in PD, it is striking that the *SNCA* gene (encoding α-SYN) has repeatedly been identified in several genome-wide association studies (GWAS)^1–3^. The importance of α-SYN in the pathogenesis of PD is highlighted by the fact that a multiplication of *SNCA* lead to severe Parkinsonism, which suggests that *SNCA* expression level, determines the severity of the PD pathology^4^.

In addition to genetic changes, understanding of epigenetic alterations is equally important to explain the dynamics of histone modifications and transcription factor binding sites that strongly regulate the gene expression. Among genetic regulators, microRNAs and transcription factors (TF) plays an essential role for regulating gene expression. Furthermore, the alterations induced by environmental factors also contribute to the disease pathology. In previous experiments our team demonstrated that decreasing methylation of intron-1 of *SNCA* increased the expression of α-SYN^5^.

Herein, we focused especially on a putative impact of the genetic variants (SNPs) distributed throughout the *SNCA* gene on the epigenetic landscape (chromatin mark diversity, nucleosome occupancy, transcription factors motifs, chromatin organization at splice sites). Furthermore, we provide structural insights into the methylation prone intron-1 region of *SNCA* as binding site of DNMT1 (DNA methyltransferase 1).

## Results

### Nucleosomes occupancy affinity for transcription factors in the vicinity of risk variants

**Figure 1:**
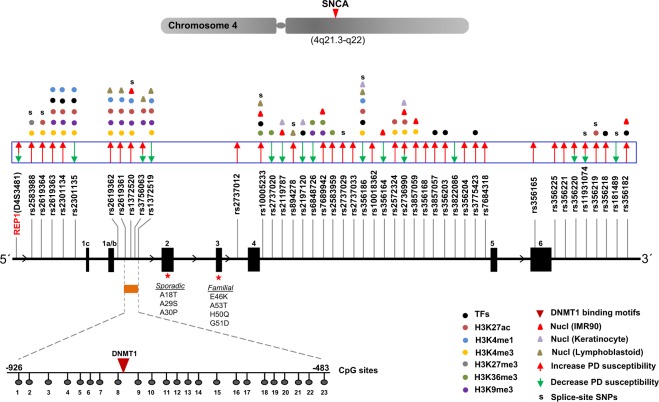
Sequence variants in *SNCA* and associated epigenetic landscape. (**A**) Figure showing exon/intron structure of *SNCA* gene at chromosome 4 of human genome. The relative positions of SNPs are plotted and high/low susceptibility of Parkinson’s disease is shown with arrows. Transcription factors and multiple histone modification marks overlays SNP sites are shown. SNPs enriched at nucleosomes are marked with triangles. DNMT1 interaction site corresponding to 23 CpG sites at intron-1 region of *SNCA* gene is demonstrated. Sporadic/familial known mutations at exon 2–3 are described for the orientation of *SNCA* gene.

**Figure 2:**
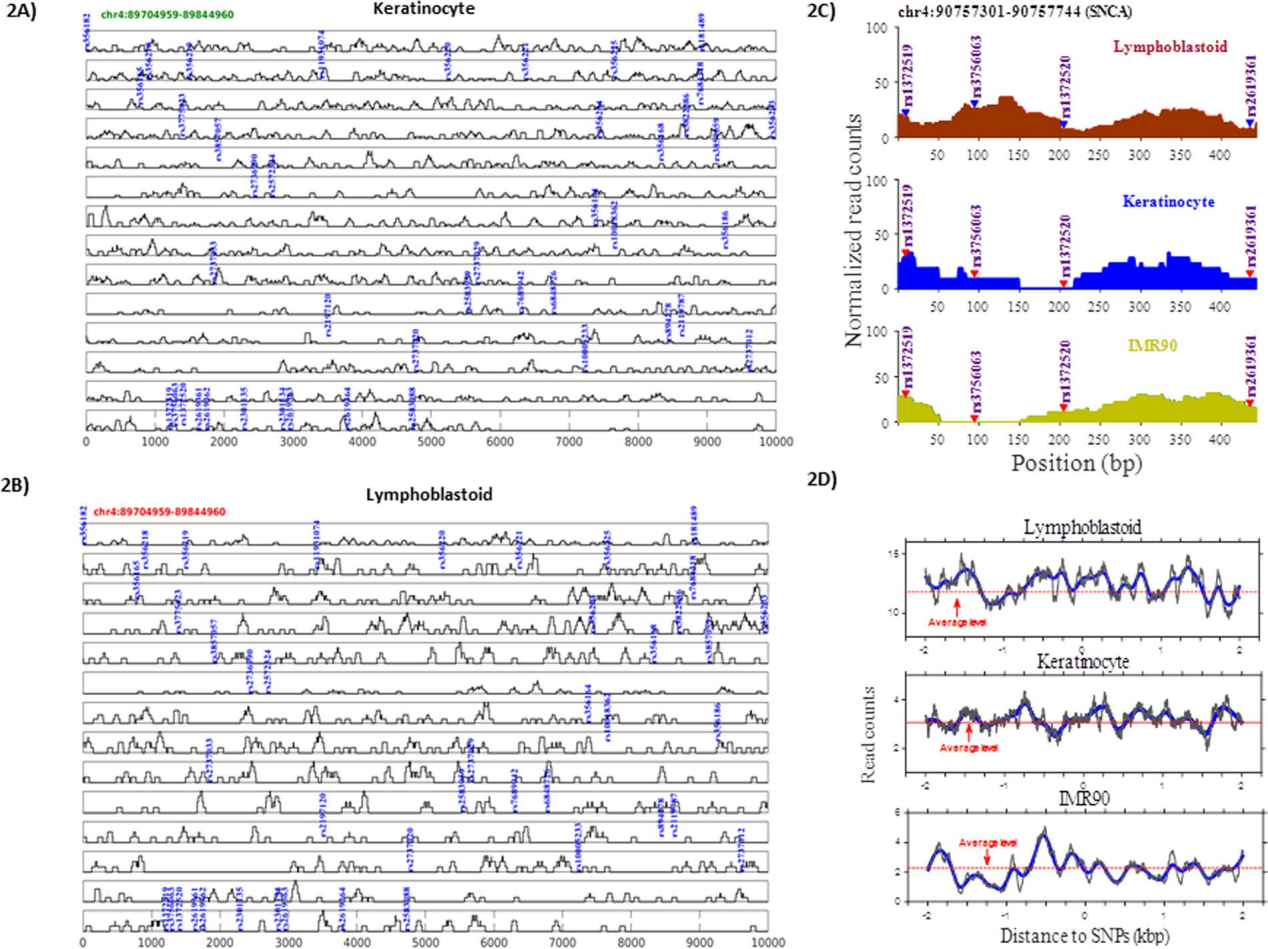
Nucleosome occupancy in vicinity of *SNCA* sequence variants. Nucleosome occupancy around the SNPs is summarized for lymphoblastoid (**A**) and keratinocytes (**B**). Nucleosome occupancy differs around the *SNCA* sequence variants (SNP sites) in three cell types with normalized read counts is drawn (**C**). Nucleosome occupancy profiles near the *SNCA* sequence variants with the profiles aligned by SNP sites and averaged by dividing number of the SNP sites is evaluated (**D**).

All the SNPs selected for this study were previously linked to increased or decreased PD susceptibility (29 SNPs: high susceptibility, 10 SNPs: low susceptibility, 4 SNPs: variability in clinical data) (Fig. 1, adapted from Campelo *et al*.^6^). The distribution of PD associated SNPs appeared to be non-random as most of them were found to be embedded in the intron 4 region. To address how the sequence variation is reflected in the occupancy profiles of nucleosomes, we categorized them on the basis of nucleosome occupancy i.e. SNPs located within nucleosomes and SNPs lacking nucleosomes in their vicinity. In order to control the processing for the sequenced datasets, we first checked the nucleosome occupancy profiles near transcription start sites (TSSs). The profiles showed a typical distribution pattern of nucleosome-depleted region (NDR) flanked by two well positioned nucleosomes, followed by equally spaced nucleosome array downstream of TSSs. The nucleosome occupancy around the selective SNPs was measured in three different cell types (keratinocytes, IMR90, lymphoblastoid; Fig. 2, Supplementary Fig. 1B). As nucleosome positioning varies in different cell types, in our analysis we show only the SNPs with significant read distribution (P ≤ 0.05) in every cell type. We also performed the analysis on undifferentiated human iPS cells (hiPS) and iPS cells differentiated to neural progenitor cells (NPC). The data clearly demonstrate that most of SNPs were located on the nucleosomes, while few among them (rs2197120, rs2737033, rs7684318) were also occupied by the nucleosome after differentiation into the NPC (Supplementary Fig. 3).

Nucleosomes impair the binding of transcription factors (TFs), thus SNPs in the TF binding sites may exert effects upon binding affinity of TFs^7,8^. Our results indicate that the effect of SNPs on TFs binding varies according to the cell types. SNP having a variable nucleosome occupancy e.g. rs1372519 was occupied in keratinocytes and IMR90, but not in lymphoblastoid (Fig. 2C,D), which also means that the SNP effect on TF binding will be in lymphoblastoid, but not in keratinocytes and IMR90. We found no correlation for genomic distance (striking periodicities across nucleosomal regions) between these SNPs. In our cumulative analysis, we identified 11 SNPs in TF binding sites, among them 5 in nucleosome positions and 3 lacked any enrichment of chromatin marks (Fig. 1, Supplementary Table 2). Among former some TFs such as FOXA1, NFATC2; Prrx2, SP1 have previously been linked with PD^9–12^.

A polymorphic microsatellite REP1 (D4S3481), located in the promoter region (~10 kb upstream) of *SNCA* also contains SNPs but they have not yet been linked to PD risk or phenotype. REP1 may however, play a role in the regulation of *SNCA* expression as its longest (263 bp) and intermediate length (261 bp) alleles are associated with an increased risk for PD^13^. Previously, the binding to transcription factor poly (ADP-ribose) transferase/polymerase-1 (PARP-1) with REP1 has been described^14^. Herein, using computationally predicted binding sites, we described additional transcription factor motifs enriched in this tandem repeats region (Fig. 1, Supplementary Fig. 2). As the Rep1 sequence contains only (TG)n and (GA)n motifs, but no CG sequences, a role for CpG-methylation can be excluded.

### Distinct chromatin structure at SNPs in different genomic regions

Enhancers and repressors, which are sometimes located far from TSSs, have been found to affect the expression of genes significantly^15–17^. In our analysis, we found SNP rs1372519 within a peak of H3K27ac (enhancer and promoter marks) and within the DNA binding motif sequence of NeuroD1 (NDF1), which had been involved in a specific neurogenic program, including differentiation and migration earlier^18^. Likewise, SNP rs2301134 is located within a peak of H3K9me3 histone modification mark (repressor and heterochromatin marks) and the DNA binding motif sequence of PRDM1 (BLIMP1), which has been shown previously to modulate the divergence of neural or germline fates through repression of SOX2 during human development^19^. SNP rs2619364 was located near a peak of H3K27me3 histone modification mark (repressor and heterochromatin marks) and a DNA binding motif sequence of RE1-Silencing Transcription factor (REST), also known as Neuron-Restrictive Silencer Factor (NRSF). NRSF is a zinc-finger transcription factor initially described as a nuclear negative regulator of differentiation^20^, now known to play a role in neuronal cells. Furthermore, we found that selective SNP sites lay within bivalent regions poised to adopt either active or repressed chromatin states. Overall, SNPs located in promotor region and intron 4 site were found to be enriched with histone modifications (H3K27ac, H3K4me1, H3K4me3, H3K27me3, H3K36me3, H3K9me3). To undermine the bivalency of histone marks, we further analyzed H3K4me1, H3K4me3, H3K36me3, H3K9me3, H3K27me3, H3K27ac and Input DNA in human monocyte, stomach, fetal muscle leg, H9 human embryonic stem cell (ESC), neural progenitor populations of neuroepithelial (NE), early radial glial (ERG) and mid radial glial (MRG). The bivalency of H3K4me3 and H3K27me3 around the transcriptional start site (TSS) of *SNCA* was observed in all tissue and cell types, while the co-existence of H3K27ac and H3K27me3 was limited to few sample types. Interestingly, the co-existence of H3K4me1 and H3K27me3 was also noticed in all the tissue and the cell types (Supplementary Fig. 4).

### Intron-1 of *SNCA*, a docking site for DNMT1

*SNCA* is expressed as distinct transcripts variants with at least three alternative transcription start sites/exons1. Previously, some activity of core promotor in the upstream of exon 1b (variant 2) was predicted^21^. But the CAGE analysis, enrichment of CpG sites and several putative transcription factor binding sites point towards an additional promotor region in the intron 1. Importantly, the methylation levels in the intron-1 of *SNCA* have been associated with expression of α-SYN in PD^5,21,22^. Therefore, we searched for potential docking sites required for putative interactions with DNMT1 in intron-1. Based on our previously published methodology, the molecular docking was conducted between the motifs identified from nucleotides and DNMT1^23^.

**Figure 3:**
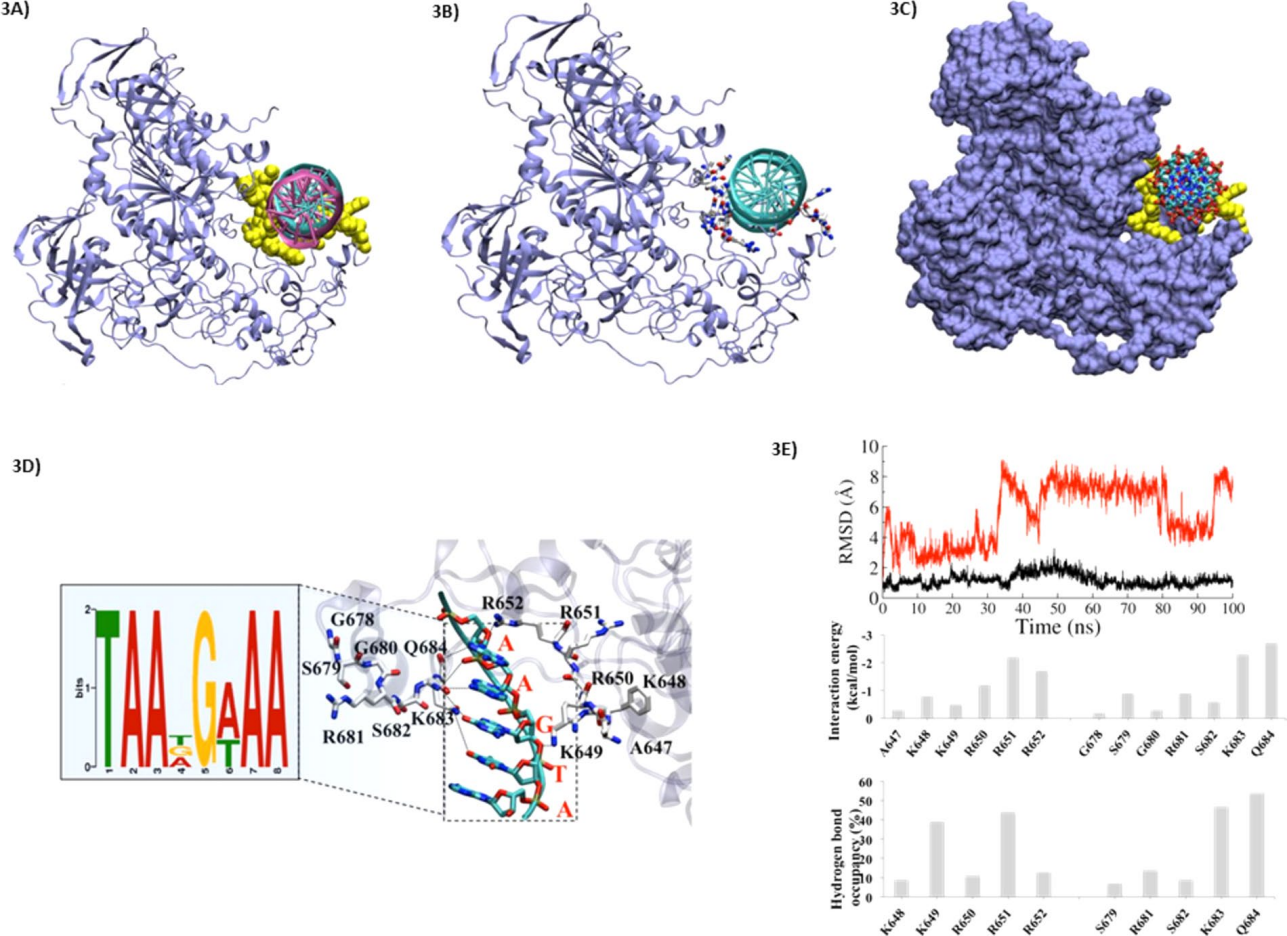
Characterization of binding site of interaction for DNMT1 with *SNCA* intron-1. (**A**) DNMT1 interaction with identified motifs (8 bp). The nucleotides are shown in cyan, and key residues are highlighted in yellow and shown in in VdW sphere. The crystalized DNA (in magenta) is shown to check the binding site. (**B**) The DNMT1 and DNA interacting zone are shown by residues shown in licorice and coded by atom wise: Carbon: white, Oxygen: red, Nitrogen: blue and Sulphur: yellow. (**C**) The DNMT1 in surface view and DNA are shown in licorice view. (**D**) The interaction site between nucleotides and key amino acids. The identified motif is highlighted in zoom-out view. (**E**) Root mean square deviation (RMSD) of APO (DNMT1 without nucleotide in red) and complex (DNMT1 with nucleotide in black) DNMT1. The dotted line is showing the average value. Residue-wise interaction energy values in kcal/mol and residue-wise hydrogen bonding occupancy of key residues in percentage wise (%) is shown.

In order to understand how the extensive positive potential created by DNMT1 influences DNA binding, we also performed rigid-body dockings with DOT (a computational docking tool for macromolecular interactions) by using our established method^24^. Docking of linear B-DNA (flexible) 8 bp from the identified motif to DNMT1 (stationary) identified the similar distinct DNA-binding sites in the 109 top-ranked conformations (Fig. 3A–C). The B-DNA conformations, which are docked (39 of the 100 solutions) showed significant interactions with the protein residues of DNMT1 protein. The side chains of highly conserved amino acids of DNMT1 (three Arginines: R650, R651 and R652) were found to be inserted into the DNA major groove, while the backbone of some other residues A647, K648, and K649 were also found to have high binding affinity. The localization of binding site constitutes the region from G678 to A685, among them the key residues which contribute heavily in the stability and high affinity of DNA motif were R681, K683 and Q684.

Because of the observed high binding affinity, we took the docked position as prime site and used it for further molecular dynamic simulations. We observed that the complementary interactions between DNMT1 and DNA fit well along with the basic patches of DNA. From the crystal structure 3SWR, it appears that the region near to DNA binding site might play a role to hold the DNA which we observed in different orientation and in different crystals. This region also shows very low electron density and highest B-factor, indicating its structural flexible.

From the crystal structure 3SWR, it appears that the missing link near DNA binding site might play a role to hold the DNA as we observed in different orientation in different crystals. This region also shows very low electron density and highest B-factor, indicating its structural flexible.

### Molecular Interaction of conserved DNA motif and DNMT1

We performed the interaction studies by using random genes VHL-150, RB1-445, WT1-390, WT1-394, and WT1-396 (including the “AGCGA” motif), according to our previously shown methodology^25^. Our data showed that the residues K649, R650, R651 and R652 make important contributions in increasing the stability of DNA-protein complexes (Fig. 3D). It was found that the key interaction of identified motif may take place with two amino acids Q684 and K683 i.e. A@Q684 and A/G/T@K683. In case of Q684 this interaction persists throughout the simulation, while K683 showed interaction with three different nucleotides. This outcome also supports the relevance of dynamics analysis rather than only static analysis through docking. From the interaction energy analysis these two interactions claimed −3.4 and −3.2 kcal/mol energy, respectively. Other key interactions were G@R650 and A@R652 (Fig. 3E, upper). The residue analysis also helps to explain the stability of the complex (Fig. 3D). The interaction energy graph revealed that 76% of binding affinity contribution is derived from the key residues. The residues A647, K648, K649, R650, R651, R652, G678, S679, G680, R681, S682, K683 and Q684 contributed > −0.5 kcal/mol. However, the maximum contribution (above ~1.5 kcal/mol) was exerted by amino acids R562, R681, K683 and Q684 (Fig. 3E, middle).

These results are supported by hydrogen bond (HB) occupancy analysis as well, in which we observed a similar trend of contribution from the residues in terms of their establishment of HBs (Fig. 3E, lower). In addition, the binding affinity of a conserved motif also showed similar kind of interaction pattern. Furthermore, the analysis from molecular dynamics also indicate that the common key residues of B-DNA (mostly G and A) interacted significantly. The results from crystal structure clearly show that the newly identified motif of nucleotides interacts with DNMT1, however, the experimental data is required to validate them. Overall, we identified two docking sites required for putative interactions with DNMT1 in intron-1, which were near to CpG8-CpG9 (Fig. 1A). It is well documented that the enzyme DNMT1 binds to DNA and adds methyl groups at the C residue of CpG islands. In this context, we can propose that the high levels of methylation at CpG8-CpG9 among 23 CpG sites observed by Jowaed *et al*.^5^ might be the result of DNMT1 interactions.

To determine DNA methylation at complete *SNCA* gene, we checked the correlation of *SNCA* expression and DNA methylation around *SNCA* in iPSC-derived dopaminergic neurons using public available data (GSE51921)^26^. The analysis showed that during the differentiation there is a slight increase in DNA methylation at upstream of *SNCA*, however, the gene expression was also increased [Fold change (PD/control) = 1.13; P-value (t-test) (log10) = 2.49; q-value = 0.053] (Supplementary Fig. 5).

## Discussion

The last few years have seen expanding knowledge of the genetic-epigenetic insights of PD pathology^27–29^. There is sufficient evidence that *SNCA*, which has been identified as first PD gene plays a central role in this disease. Therefore, in this study, we focused on the PD associated SNPs embedded within *SNCA* gene and investigated their epigenetic landscape. Herein, we could show that the risk-associated SNPs are enriched at the nucleosomes and are associated with multiple histone modifications. Among repressive histone marks (H3K27me3, H3K9me3), H3K9me3 was uniformly present in most SNP sites located at promoter region, while H3K27me3 correlates only to rs2583988. Similarly, histone marks for active chromatin (H3K27ac, H3K4me1, H3K4me3, H3K36me3) were also found to be overlapping with certain variants. In intron 4 specifically, we could define 2 regions (Chr4:89821930–89800236; 89772075–89753230), where most of PD related SNPs were located. How the interplay of active/repressive histone marks contributes towards *SNCA* regulation through these SNP sites is unclear. However, previously studies provided a hint that the genetic variants might engage in crosstalk with sequence-specific transcription factors and epigenetic patterns (including DNA methylation^30,31^) thereby impacting the transcription of genes locally or remotely^32,33^. Importantly, each histone modification is unique to regulate the predictive chromatin signature at promoters, enhancer and TSS, still they can also share overlapping chromatin^34^ signatures^35^. This is what we have observed in several SNP sites, where bivalent signature (co-existing “activating” and “silencing” nucleosomal modifications) poised to adopt either active or repressed chromatin states. It is noteworthy to mention that even though the bivalency of H3K4me3 and H3K27me3 around the transcriptional start site (TSS) of SNCA was observed in all tissue and cell types, the low SNCA expression was also observed in some samples (e.g. stomach and fetal muscle). Thus, it can be argued that the observed bivalency at the given locus simply reflects the cellular heterogeneity (mosaic signal from the mixed population of cells). Another possibility might be the repression of SNCA antisense overlapping with SNCA (at their 5′ ends) by H3K27me3 histone mark. Therefore, in the future, the emerging single-cell epigenomic methods hold the potential to add deeper insights into SNCA gene regulation.

Apart from histone medications, disease variants are also known to alter transcriptional levels. Specifically, the variants in coding regions may confer disease risk through altered protein sequences, however, variants in non-coding regions or regulatory variants (enhancer, sequence/TF binding sites)^36–38^ can also contribute to disease susceptibility by changing the gene transcription^39^. Brenner *et al*. identified and confirmed the bindings of two zinc finger proteins ZSCAN21 (intron-1) and GATA2 (intron-2) in *SNCA*, but did not observe any SNP or mutations within these binding sites^40^. Recently, two RNA-binding proteins (ELAVL1, TIAR) that target *SNCA* 3′UTRs and controlling its expression were also discussed^41^. Herein, we specifically investigated TF motifs overlapping the SNPs and found that 11 SNPs shared the affinity towards specific TFs. Interestingly, 2 of them were splice site SNPs and rs356186 among them emerged as the one with preferential sites for TFs as well as for various chromatin modifications. We could also show many of these TFs (FOXA1, NFATC2; Prrx2, SP1) have previously been implicated in PD. We extended our analysis for repetitive microsatellite D4S3481 (called REP-1) located upstream of *SNCA* gene and found several associated TFs motifs in and around the repetitive sequences. Recently, Afek *et al*. claimed that two TF families (GATA and ELK) have consensus binding sites in a very close proximity to REP-11 region, which appears to be potentially relevant for *SNCA* transcriptional regulation^42^. Till date, apart from the expansion of repetitive sequence, none of the SNP inside this locus have been proven to be predictive for PD. Previously, It was shown that the alternative splicing also contribute towards the dysregulation of SNCA^43^, therefore, our findings of chromatin regulation at splice site SNPs provide useful insights towards the molecular characteristics of *SNCA* gene.

To evaluate further evidence for genetic-epigenetic regulation of *SNCA* gene, we investigated promoter specific CpG rich region overlapping these SNP sites. We identified a DNMT1 binding site specifically at CpG8-CpG9 region of intron-1 region of *SNCA*, where a high level of methylation was previously observed by Jowaed *et al*.^5^. The control of DNMTs in SNCA locus is also confirmed by one recent study in which authors by using CRISPR-deactivated Cas9 (dCas9) fused with the catalytic domain of DNA-methyltransferase 3A (DNMT3A) claimed to reduce the expression levels of *SNCA* in human-induced pluripotent stem cell (hiPSC)–derived dopaminergic neurons from a PD patient with *SNCA* triplication^44^. Therefore, we propose that the sequence specific recognition of DNMT1 we identified in this study may impose the intrinsic control of *SNCA* specific gene regulation. Apart from DNMT1, other unknown factor(s) may also have an additional role in *SNCA* regulation e.g. some risk variant in a non-coding distal enhancer element were predicted to regulate the expression of α-synuclein^45^. Therefore, we checked the correlation of *SNCA* expression and DNA methylation in iPSC-derived dopaminergic neurons and observed an increase in both DNA methylation and expression at this particular locus. Hence, the contribution of multiple factors towards the expression of *SNCA* cannot be excluded. On a broader view, the multiplication of *SNCA* gene, hypomethylation of *SNCA* intron1, the A53T mutation and mutation of miRNA binding sites in the 3′UTR of *SNCA*, at least in part, may participate in the regulatory mechanisms of *SNCA* expression.

Taken together, we report an in-depth characterization of the epigenetic landscape of PD linked risk polymorphisms distributed across *SNCA* gene. The blueprint (Fig. 1: increased/decreased PD susceptibility) we have presented here will help to determine the clinical outcome of newly screened patients. Our data suggest that the chromatin marks overlaying the disease associated SNPs in *SNCA*, might alter the genomic architecture and contribute towards the disease susceptibility. Hence, on molecular level, we pose an open question, whether these disease risk variants can disproportionately influence the gene expression among different tissues/anatomical regions of the brain to cause phenotypic heterogeneity in PD.

## Material and Methods

### Classification and retrieval of SNPs

We investigated the panel of 34 SNPs previously found in GWAS studies and from the literature in association to *SNCA* gene and PD susceptibility. The genomic locations were retrieved from dbSNP database (http://www.ncbi.nlm.nih.gov/SNP/) and NCBI for computational analysis. For predicting the splice acceptor or donor sites and exonic enhancer and silencer elements, we used Human Splicing Finder tool (http://www.umd.be/HSF3/HSF.shtml). The input sequence used was 50 bp up- and downstream of the SNP position related to the disease associated ten rsIDs. From the output, only predictions with score more than 70 percent were considered in results. Transcription factor enrichment analysis was performed by using SNP2TFBS (http://ccg.vital-it.ch/snp2tfbs/) tool which selects and visualizes user defined variants that affect single or multiple transcription factors. Apart from *SNCA* gene, Rep1 (Accession no. U46895; D4S3481), the polymorphic microsatellite repeat located approximately 10 kb upstream of the *SNCA* gene, was also checked for 9 SNP sites and TF binding sites.

### Histone modifications and regulatory SNPs in the *SNCA*

To examine the overlap between SNPs and histone modification marks, FASTQ files of ChIP-seq data of H3K27ac, H3K4me1 and Input DNA in neuron were download from Sequence Read Archive (SRA) database referred from Gene Expression Omnibus (GEO) database (GSE71278), FASTQ files of ChIP-seq data of H3K4me3, H3K36me3, H3K9me3, H3K27me3 and Input DNA in fetal brain (GSE17312; GSM621457, GSM621410, GSM621393, GSM621427, GSM916054, GSM706851) were downloaded respectively. BAM files of ChIP-seq data of H3K4me1, H3K4me3, H3K36me3, H3K9me3, H3K27me3, H3K27ac and Input DNA in human monocyte, stomach, fetal muscle leg (ENCSR518BPP, ENCSR949WGV, ENCSR820MXK) were downloaded respectively from ENCODE project database (https://www.encodeproject.org). FASTQ files of ChIP-seq data of H3K27ac, H3K27me3, H3K4me1, H3K4me3 and Input DNA in H9 human embryonic stem cell (ESC) and neural progenitor populations of neuroepithelial (NE), early radial glial (ERG) and mid radial glial (MRG) were downloaded from SRA database referred from GEO database (GSE62193). Reads in FASTQ files of ChIP-seq data were aligned to the hg19 version of the human reference genome using BWA mem with default parameter^46^. BAM files produced by BWA were converted to SAM files, sorted and indexed by Samtools^47^. Peak calling of ChIP-seq data was performed by SICER with optional parameters ‘hg19 1 200 150 0.74 600 0.01’^48^. WIG files of ChIP-seq data were produced from the BAM files by bam2wig.pl with optional parameters ‘–extend–rpm–bin 200’ in Biotoolbox (https://github.com/tjparnell/biotoolbox), and were displayed using custom truck in UCSC Genome browser (http://genome.ucsc.edu/). The overlaps between SNPs and peaks of histone modification marks were investigated using intersectBed of Bed tools with default parameter^49^. In context to check the overlap between SNPs and open chromatin regions, we obtained the Bed file of peaks of DNase-DGF data in fetal brain from NIH Epigenome roadmap website (http://www.roadmapepigenomics.org and GEO database (GSM723021), and investigated the overlaps between SNPs and peaks of open chromatin regions using intersect Bed.

Repeat DNA sequences were searched for hg19 version of the human reference genome using RepeatMasker (http://www.repeatmasker.org) and RepBase RepeatMasker Edition (http://www.girinst.org). The overlap of repeat DNA sequences and SNPs was examined using intersectBed.

### Nucleosome positioning around single nucleotide polymorphisms

To determine the nucleosome occupancy around SNPs, sequencing datasets of nucleosomes were retrieved from three cell types: lymphoblastoid (Gene expression omnibus (GEO) accession number: (GSM907783), keratinocytes (GSE65191), IMR90 (GSE44985) and neural progenitor cells (GSE117870). Reads in the datasets were determined with micrococcal nuclease digestion followed by next generation sequencing (MNase-Seq). Coordinates of single nucleotide polymorphisms (SNP) sites and transcription start sites (TSSs) were retrieved from UCSC using the Tables function (http://genome.ucsc.edu) in human genome assemble hg19 (GRCh7.p13). For nucleosome occupancy profile, the raw reads was mapped to human genome (hg19) using Bowtie^50^. Only the uniquely mapped reads were used to further analysis. Nucleosome occupancy refers to reads count at each genomic locus which means the count of the reads covering the locus. Each read was extended to 73 bp in the 3′ direction and shifted by 36 bp towards the 3′ direction. The nucleosome occupancy profiles near special sites (e.g., SNP sites) were represented with the average nucleosome occupancy profile, which was calculated by summing the occupancy signal at each genomic site and then dividing the summed signal by the gene number. For the SNPs, the raw reads count data was normalized by dividing by the maximal reads count of whole genome and then multiplying by 100.

Significance of reads enrichment at each SNP sites was calculated with Poisson distribution

($P(k)={e}^{-\lambda }{\lambda }^{k}/k!$, λ indicates the average reads count, *k* is reads count at SNP sites). A SNP site with a P ≤ 0.05 was considered as being occupied by nucleosomes.

### DNMT1-SNCA Intron-1 binding analysis

The intron-1 region of *SNCA*, previously evaluated for DNA methylation levels was used to find DNMT1 interacting sites^5^. The identification of motifs from nucleotide sequences, molecular docking and molecular dynamics simulation was performed according to the methods we have published previously^23–25^.

## Supplementary information

Supplementary info

Supplementary Data
