# Supplementary material for: Common genetic variants associated with Parkinson’s disease display widespread signature of epigenetic plasticity
Source: Sci Rep. 2019 Dec 5;9:18464. doi: 10.1038/s41598-019-54865-w (PMC6895091; doi:10.1038/s41598-019-54865-w)
Supplement: Supplementary file 1 — Supplementary info [file 41598_2019_54865_MOESM1_ESM.docx]

### Common genetic variants associated with Parkinson’s disease display widespread signature of epigenetic plasticity

Amit Sharma, Naoki Osato, Hongde Liu, Shailendra Asthana, Tikam Chand Dakal, Giovanna Ambrosini, Philipp Bucher, Ina Schmitt, Ullrich Wüllner

**Supplementary info**

**Supplementary Figure 1: Evaluation of data quality for nucleosome DNA sequence data and nucleosome occupancy profile near *SNCA* sequence variants**

A) Nucleosome occupancy profiles in vicinity of transcription start sties (TSSs) in the three cell types showing the expected patterns. **B)** Nucleosome occupancy profile around *SNCA* sequence variants in cell type IMR90 is shown.

**Supplementary Figure 2:** Transcription factor binding affinity of *SNCA*-Rep1 microsatellite

**Supplementary Figure 3: Nucleosome occupancy in neural progenitor cells**

The data comprised of undifferentiated human iPS cells (hiPS) and iPS cells differentiated to neural progenitor cells (NPC) is shown. The original occupancy signal normalized by dividing the average reads count (first column) and the nucleosome occupancy region identified by calling the peaks for the nucleosome occupancy signal (second column) are estimated. SNPs located on the nucleosome are marked in circle.

**Supplementary Figure 4: Determination of histone bivalents in multiple samples**

Chip-seq data for H3K4me1, H3K4me3, H3K36me3, H3K9me3, H3K27me3, H3K27ac and Input DNA in human monocyte, stomach, fetal muscle leg, H9 human embryonic stem cell (ESC), neural progenitor populations of neuroepithelial (NE), early radial glial (ERG) and mid radial glial (MRG) is shown.

**Supplementary Figure 5: Differential DNA methylation level in *SNCA* gene**

DNA methylation level between iPSC-derived dopaminergic neurons (PD) and healthy subjects (Non-PD) retrieved from publically available data (GSE51921) is shown.

**Supplementary Table 1**: PD associated splice site SNPs in *SNCA* gene

**Supplementary Table 2:** Transcription factor binding affinities of *SNCA*-SNPs
