# Supplementary material for: Common genetic variants associated with Parkinson’s disease display widespread signature of epigenetic plasticity
Source: Sci Rep. 2019 Dec 5;9:18464. doi: 10.1038/s41598-019-54865-w (PMC6895091; doi:10.1038/s41598-019-54865-w)
Supplement: Supplementary file 2 — Supplementary Data [file 41598_2019_54865_MOESM2_ESM.pdf]

Supplementary information

Common genetic variants associated with Parkinson's disease display widespread signature of epigenetic plasticity

Amit Sharma, Naoki Osato, Hongde Liu, Shailendra Asthana, Tikam Chand Dakal, Giovanna Ambrosini, Philipp Bucher, Ina Schmitt, Ullrich Wüllner

Supplementary Figure 1: Evaluation of data quality for nucleosome DNA sequence data and nucleosome occupancy profile near *SNCA* sequence variants

1A)

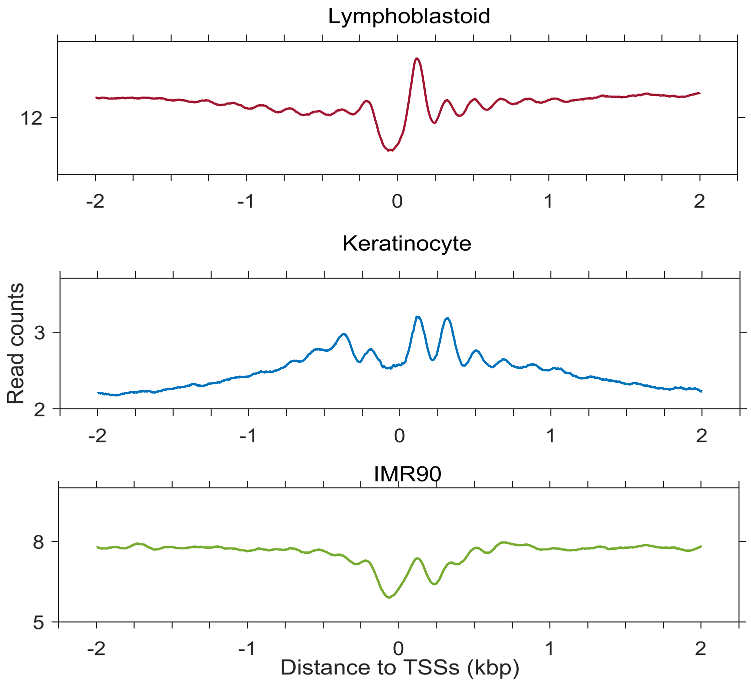

1B)

IMR90

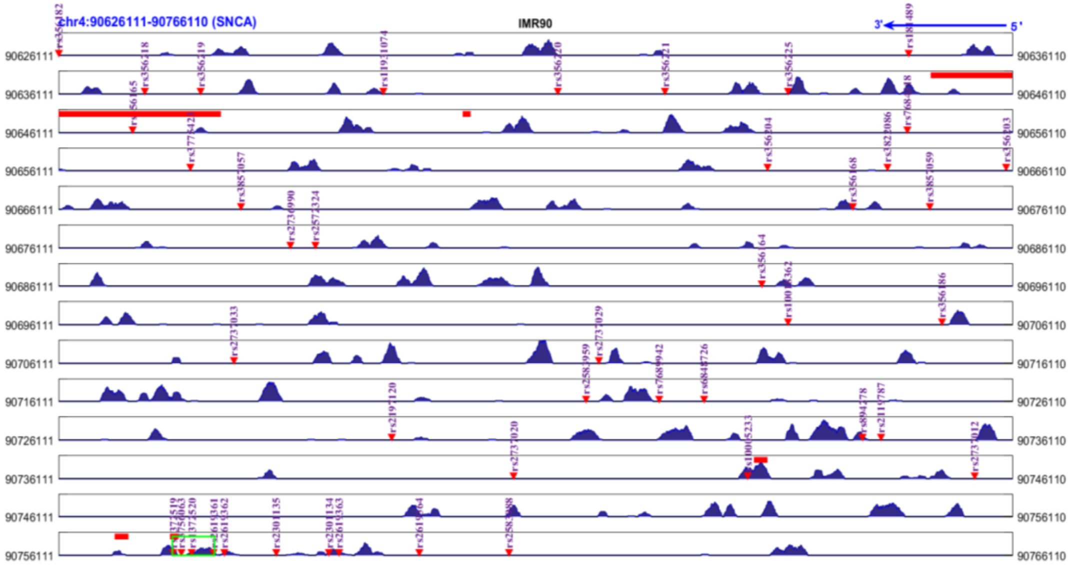

Supplementary Figure 2: Transcription factor binding affinity of *SNCA*-Rep1 microsatellite

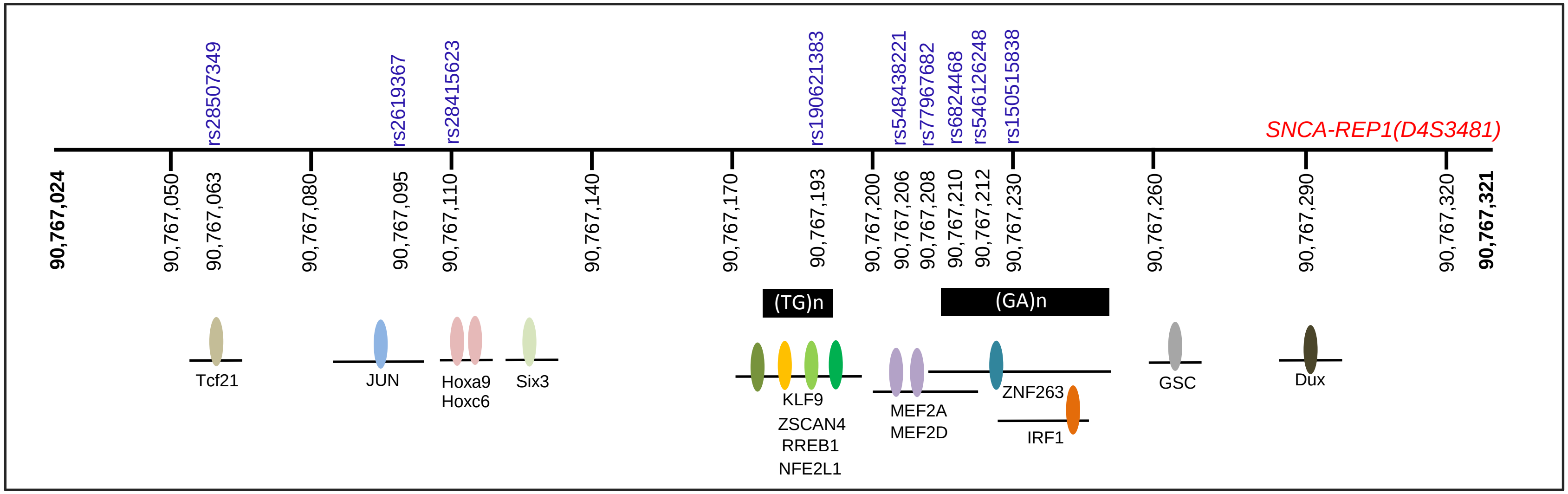

Supplementary Figure 3: Nucleosome occupancy in neural progenitor cells

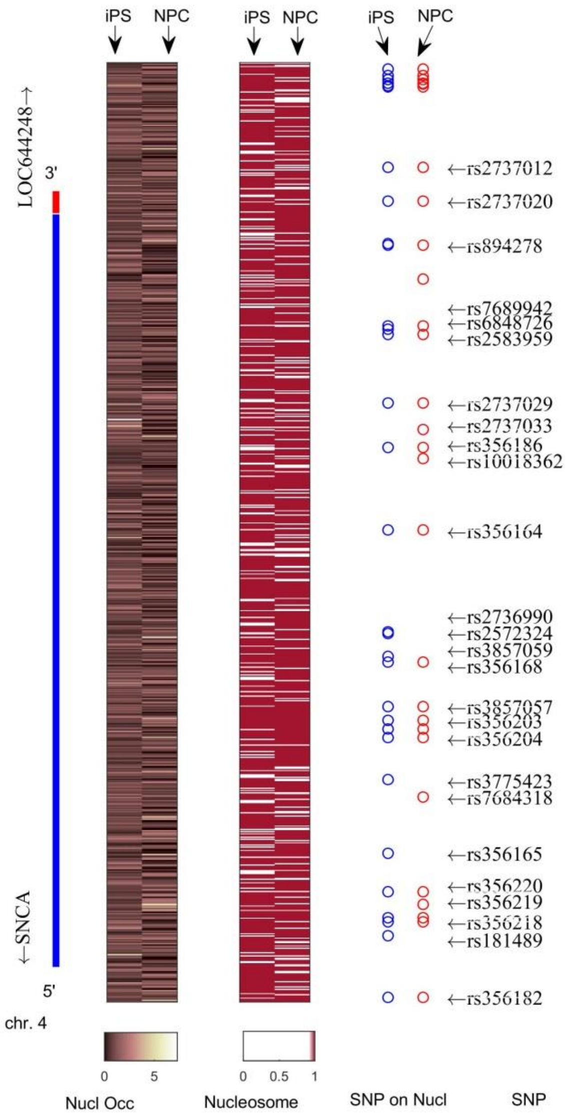

Supplementary Figure 4: Determination of histone bivalents in multiple samples

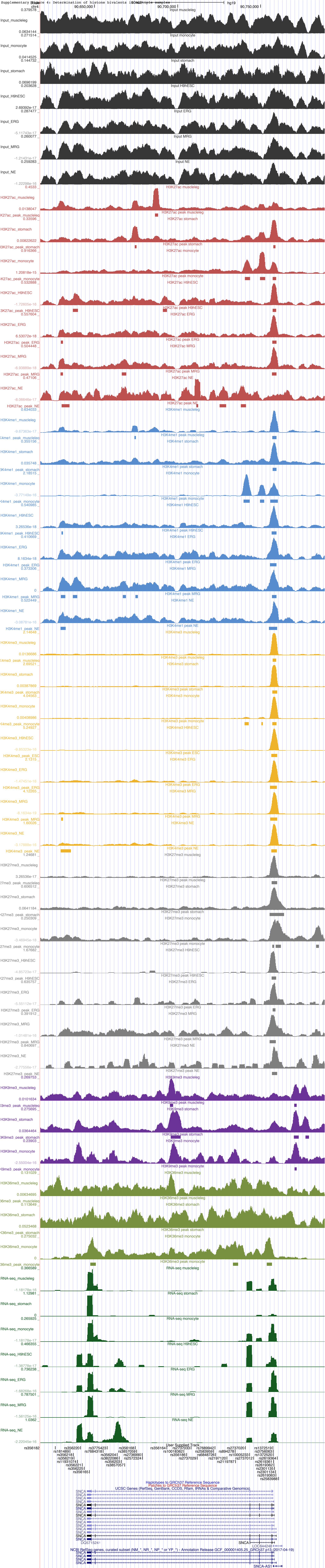

Supplementary Figure 5: Differential DNA methylation level in *SNCA* gene.

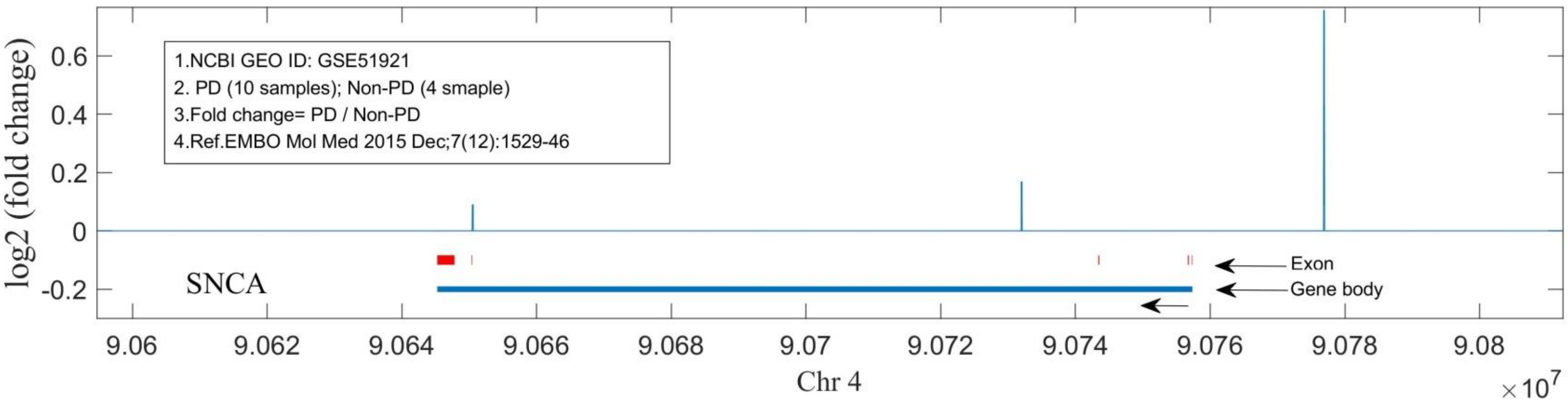

Supplementary Table 1: PD associated splice site SNPs in *SNCA* gene

S.No.	rsIDs	GRCh38/hg38	Codon	Splice site prediction and type	Motif type	Pattern	Score	Reference
1	rs181489	89713869	T (REF)	Enhancer	SRp55	TGAGGA	74,82%	ESE-Finder
				Enhancer	SF2/A SF	ATGAGGA	73,85%	ESE-Finder
			C (ALT)	Loss of Enhancers	Srp55 and SF2/A SF	-	-	-
			A (ALT)	Loss of Enhancer	SRp55	-	-	-
				Gain of Silencer	ESS#1	AAAGAGGA	83,57	Sironi et al., 2004
2	rs356219	89716450	G (REF)	Enhancer	SRp40	TTAGACG	85,99%	ESE-Finder
				Enhancer	SF2/A SF	TAGACGT	79,32%	ESE-Finder
			A (ALT)	Loss of Enhancer	SF2/A SF	-	-	-
3	rs11931074	89718364	G (REF)	Enhancer	SRp40		93,59	ESE-Finder
				Enhancer	SC35		79,04	ESE-Finder
				Silencer	ESS #2		83,2	Sironi et al., 2004
				Silencer	ESS #1		75,98	Sironi et al., 2004
			T (ALT)	Loss of Enhancer and Silencer sites	SC35 and ESS#1	-	-	-
			C (ALT)	Loss of Silencer	ESS#1	-	-	-
				Gain of Enhancer	SRp40	CTGCAGG	80,36	ESE-Finder
				Gain of Enhancer	SRp55	TGCAGG	75,65	ESE-Finder
			A (ALT)	Loss of Silencer	ESS#1	-	-	-
				Gain of Enhancer	SF2/A SF (IgM)	CTGAAGG	73,54	ESE-Finder
4	rs356186	89784213	A (REF)	Enhancer	SRp40	AGAAAGG	79,4	ESE-Finder
				Enhancer	SF2/A SF	GAAAGGA	73,79	ESE-Finder
			G (ALT)	Loss of Enhancer	SRp40	-	-	-
				Gain of Enhancer	SFA/A SF(IgM)	CAGATGA	80,92	ESE-Finder
				Gain of Silencer	ESS#2	AGAAGGGA	75,13	Sironi et al., 2004
5	rs2737029	89790619	T (REF)	Acceptor site	-	tggttcccccagTG	86,86	-
				Donar site	-	ATGgttccc	66,28	-
			C (ALT)	No change in Acceptor or Donar site	-	-	-	-
6	rs894278	89813384	T (REF)	Enhancer	SRp40	TCAAATG	78,5	ESE-Finder
			G (ALT)	No change in Enhancer site	-	-	-	-
7	rs10005233	89822180	C (REF)	Enhancer	SFA/A SF(IgM)	CTCACAT	79	ESE-Finder
				Enhancer	SF2/A SF	CTCACAT	76,47	ESE-Finder
				Enhancer	SC35	GGCCTCAC	75,17	ESE-Finder
				Silencer	ESS#3	CCTCACAT	75,37	Sironi et al., 2004
			T (ALT)	Loss of Enhancers	SFA/A SF(IgM), SF2/A SF and SC35	-	-	-
8	rs2619363	89837896	G (REF)	Acceptor site	-	agcgcctgccagAC	80,93	-
				Enhancer	SFA/A SF(IgM)	CGCCTGC	72	ESE-Finder
			T (ALT)	No change in Acceptor	-	-	-	-
				Loss of Enhancer	SFA/A SF(IgM)	-	-	-
				Gain of Enhancer	SC35	AGCTCCTG	87,83	ESE-Finder
			C (ALT)	No change in Acceptor	-	-	-	-
				Loss of Enhancer	SFA/A SF(IgM)	-	-	-
				Gain of Enhancer	SC35	AGCTCCTG	89,24	ESE-Finder
9	rs2619364	89838736	A (REF)	Acceptor site		cagagcaagaagGT	69	-
				Enhancer	SC35	GACCAGAG	83,04	ESE-Finder
				Enhancer	SRp40	CCAGAGC	82,46	ESE-Finder
				Enhancer	SF2/A SF	CAGAGCA	80,02	ESE-Finder
				Silencer	ESS#1	CCAGAGCA	79,23	Sironi et al., 2004
			G (ALT)	No change in Acceptor	-	-	-	-
				Loss of Enhancer	SRp40	-	-	ESE-Finder
				Gain of Enhancer	SFA/A SF	CGGAGCA	77,4	ESE-Finder
				Gain of Enhancer	SFA/A SF(IgM)	CGGAGCA	82,77	ESE-Finder
				Gain of Enhancer	SFA/A SF(IgM)	GACCGGA	70,69	ESE-Finder
			C (ALT)	No change in Acceptor	-	-	-	-
				Loss of Enhancer	SRp40	-	-	-
				Gain of Enhancer	SC35	GGACCCGA	76,77	ESE-Finder
				Gain of Enhancer	SFA/A SF(IgM)	CCGAGCA	75,31	ESE-Finder
				Gain of Enhancer	SFA/A SF(IgM)	GACCCGA	72,31	ESE-Finder
10	rs2583988	89839677	C (REF)	No site found	-	-	-	-
			T (ALT)	Gain of Enhancer	SRp55	TACTTC	77,45	ESE-Finder
			A (ALT)	No site found	-	-	-	-

Supplementary Table 2: Transcription factor binding affinities of *SNCA*-SNPs

rs356182	chr4	90626110	90626111	G	A	MATCH=5;TF=Foxa2,FOXP2,FOXA1,FOXP1,FOXO3;ScoreDiff=173,114,95,52,0
rs356218	chr4	90637009	90637010	A	G	MATCH=1;TF=NFATC2;ScoreDiff=0
rs3775423	chr4	90657490	90657491	C	T	MATCH=1;TF=ARID3A;ScoreDiff=0
rs356203	chr4	90666040	90666041	C	T	MATCH=1;TF=ARID3A;ScoreDiff=0
rs3857057	chr4	90668018	90668019	A	G	MATCH=1;TF=Hand1_Tcfe2a;ScoreDiff=-28
rs356168	chr4	90674430	90674431	G	A	MATCH=2;TF=Pdx1,Prrx2;ScoreDiff=0,0
rs2197120	chr4	90729601	90729602	A	G	MATCH=1;TF=Sox5;ScoreDiff=0
rs10005233	chr4	90743330	90743331	C	T	MATCH=1;TF=TFAP2C;ScoreDiff=-104
rs2301135	chr4	90758388	90758389	G	C	MATCH=3;TF=SP1,SP2,KLF5;ScoreDiff=255,165,98
rs2301134	chr4	90758944	90758945	A	G	MATCH=1;TF=PLAG1;ScoreDiff=281
rs2619363	chr4	90759046	90759047	G	T	MATCH=1;TF=NFYA;ScoreDiff=-36
